# Single-Step Fabrication of BiOI Nanoplates as Gas Diffusion Electrodes for CO_2_ Electroreduction to Formate: Effects of Spray Pyrolysis Temperature on Activity and Flooding Propensity

**DOI:** 10.1021/acsanm.4c02570

**Published:** 2024-07-24

**Authors:** Kornkamon Meesombad, Kasempong Srisawad, Pongtanawat Khemthong, Teera Butburee, Chattarika Sukpattanacharoen, Kajornsak Faungnawakij, Pongkarn Chakthranont

**Affiliations:** † National Nanotechnology Center (NANOTEC), National Science and Technology Development Agency (NSTDA), Khlong Luang, Pathum Thani 12120, Thailand; ‡ Division of Innovation and Research, Department of Disease Control, Ministry of Public Health, Nonthaburi 11000, Thailand

**Keywords:** electrochemical CO_2_ reduction, BiOI, Bi_2_O_2_CO_3_, formate, sol−gel fabrication, gas diffusion, flooding

## Abstract

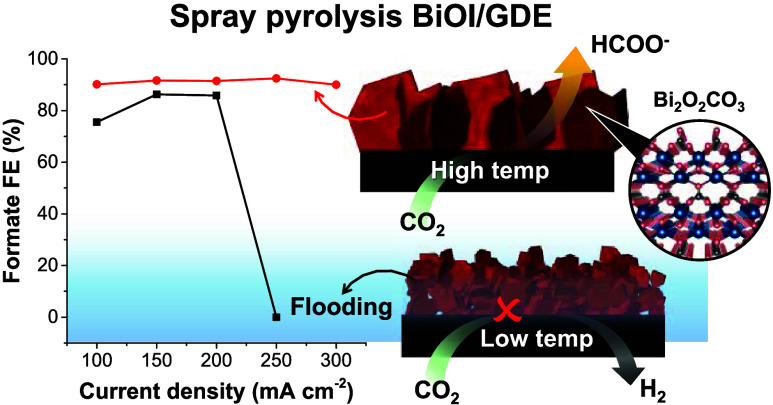

Bismuth-based electrocatalysts for carbon dioxide (CO_2_) reduction are notable for their high formate selectivity, scalability, affordability, and low toxicity. Here, we introduced a facile spray pyrolysis method to fabricate catalyst-coated gas diffusion electrodes (GDE) in one step. Our study revealed that deposition temperatures significantly affected the morphology, crystal orientation, and impurity of bismuth oxyiodide (BiOI) nanoplates. Specifically, BiOI prepared at 250 °C (BiOI-250) exhibited exceptional Faradaic efficiency (>90%) for formate production at a high current range (100–300 mA cm^–2^) and demonstrated outstanding stability (>30 h). In situ Raman spectroscopy indicated that BiOI-250’s superior performance stemmed from its resilience to microscopic flooding, a failure mechanism observed in low-temperature BiOI. X-ray absorption spectroscopy (XAS) showed that BiOI-250 predominantly consisted of the active Bi_2_O_2_CO_3_ phase, while low-temperature BiOI contained a mixture of Bi_2_O_2_CO_3_ and the less active Bi metal, formed via the reduction of the Bi_2_O_3_ impurity. This impurity led to increased catalyst resistivity, uneven potential distribution, and restructuring, contributing to flooding. Our study underscores the crucial role of catalyst structures in determining electrode performance and flooding propensity, offering key insights for optimizing bismuth-based electrocatalysts for CO_2_ reduction.

## Introduction

Electrochemical carbon dioxide reduction (CO_2_RR) has emerged as a promising technology for carbon utilization, driven by the urgent need to address the irreversible impacts of the climate crisis. This CO_2_ conversion process not only operates effectively under mild conditions, utilizing water and electricity as primary inputs, but also produces a variety of valuable products, including carbon monoxide (CO), methane (CH_4_), ethanol (C_2_H_5_OH), ethylene (C_2_H_4_), and formic acid (HCOOH), contingent upon the catalyst employed.^1^ Formic acid stands out as the most economically viable CO_2_RR product due to its high market price and low energy consumption as it requires only 2-electron transfer per molecule.^2^ Most importantly, high selectivity of the CO_2_RR to formate has already been demonstrated using non-noble catalysts such as tin (Sn), lead (Pd), cadmium (Cd), indium (In), and bismuth (Bi).^3^

Bi-based electrocatalysts are particularly promising candidates for large-scale formate production, owing to their superior selectivity, cost-effectiveness, and low toxicity.^4^ Previously reported high-performance Bi-based catalysts encompass a diverse range of compounds and nanostructures, such as Bi metal,^5,6^ Bi oxide,^7−10^ Bi oxyhalides,^11,12^ Bi sulfides,^13,14^ Bi-based metal–organic frameworks (MOFs),^15^ Bi phosphates,^16^ Bi nanowires,^17−19^ Bi nanosheets,^20,21^ and Bi nanoclusters.^16,22,23^ These catalysts consistently exhibited high formate Faradaic efficiency, surpassing 70%, and maximum current density ranging from tens of mA cm^–2^ to almost 2 A cm^–2^ (Table S1).^24−28^

Among the highly active catalysts, Bi oxyhalides, denoted as BiOX (X = I, Cl, Br), have received considerable attention due to their well-defined layered structure, which can be produced through scalable and straightforward synthesis methods.^29^ BiOX is characterized by layers composed of (Bi_2_O_2_)^2+^ slabs separated by double slabs of halogen ions into a sequence of [X - Bi - O - Bi -X] arrangements through nonbonding van der Waals interactions along the *c*-axis or [001] direction.^30,31^ This layer structure leads to anisotropic electron effective masses, resulting in higher conductivity along the in-plane direction compared to across the planes.^32,33^ During the CO_2_RR, halide ions between the Bi–O interlayer rapidly exchange with CO_3_^2–^ ions, forming the active Bi_2_O_2_CO_3_ phase. BiOX electrocatalysts, irrespective of the halide type, have shown promising selectivity for formate production.^12,34^ Nevertheless, these catalysts still encounter limitations, including low catalytic activity and poor stability.

The anisotropic nature of BiOX introduces the potential for manipulating material morphologies, particularly the dimensions of crystal facets and crystal orientation, thereby impacting the CO_2_RR activity for formate production. Distinct facet exposures may alter CO_2_ adsorption energy and the rate of formate intermediate formation,^12,35^ while crystal orientation can enhance the catalyst’s conductivity and stability. These morphological features are influenced by catalyst synthesis and electrode fabrication processes. Typically, a hydrothermal method is employed to synthesize BiOX powder, subsequently transformed into a catalyst ink and spray coated onto a carbon substrate.^36,37^ This approach provides precise control over the crystal morphology at the expense of crystal orientation concerning the substrate. Deposition of catalyst powder with a random orientation may result in tortuous conductive pathways and poor adhesion, potentially leading to reduced activity and stability.

In this study, we investigated the efficacy of a spray pyrolysis method for directly growing BiOX nanoplates, particularly BiOI, onto a gas diffusion layer (GDL) in a single step. BiOI was selected over BiOCl and BiOBr as it releases the least corrosive halogen gas during the synthesis. We discovered that the morphology and orientation of the BiOI nanoplates could be manipulated by adjusting the deposition temperature. It was discovered that a higher deposition temperature resulted in pure-phase BiOI nanoplates oriented perpendicular to the substrate, forming a continuous conductive pathway along the in-plane direction. Hence, BiOI nanoplates synthesized at a higher temperature exhibited higher activity and resilience to flooding than those synthesized at lower temperatures, as characterized by *in situ* Raman spectroscopy. This spray pyrolysis technique offers a streamlined, scalable, single-step electrode preparation process that allows precise control of BiOI morphology and orientation, resulting in a high-performing CO_2_RR electrode with excellent selectivity for formate (>90%) and good stability (>30 h), on par with the state-of-the-art Bi-based catalysts reported in the literature (Table S1).

## Materials and Methods

### Catalyst Preparation and Characterization

**Figure 1:**
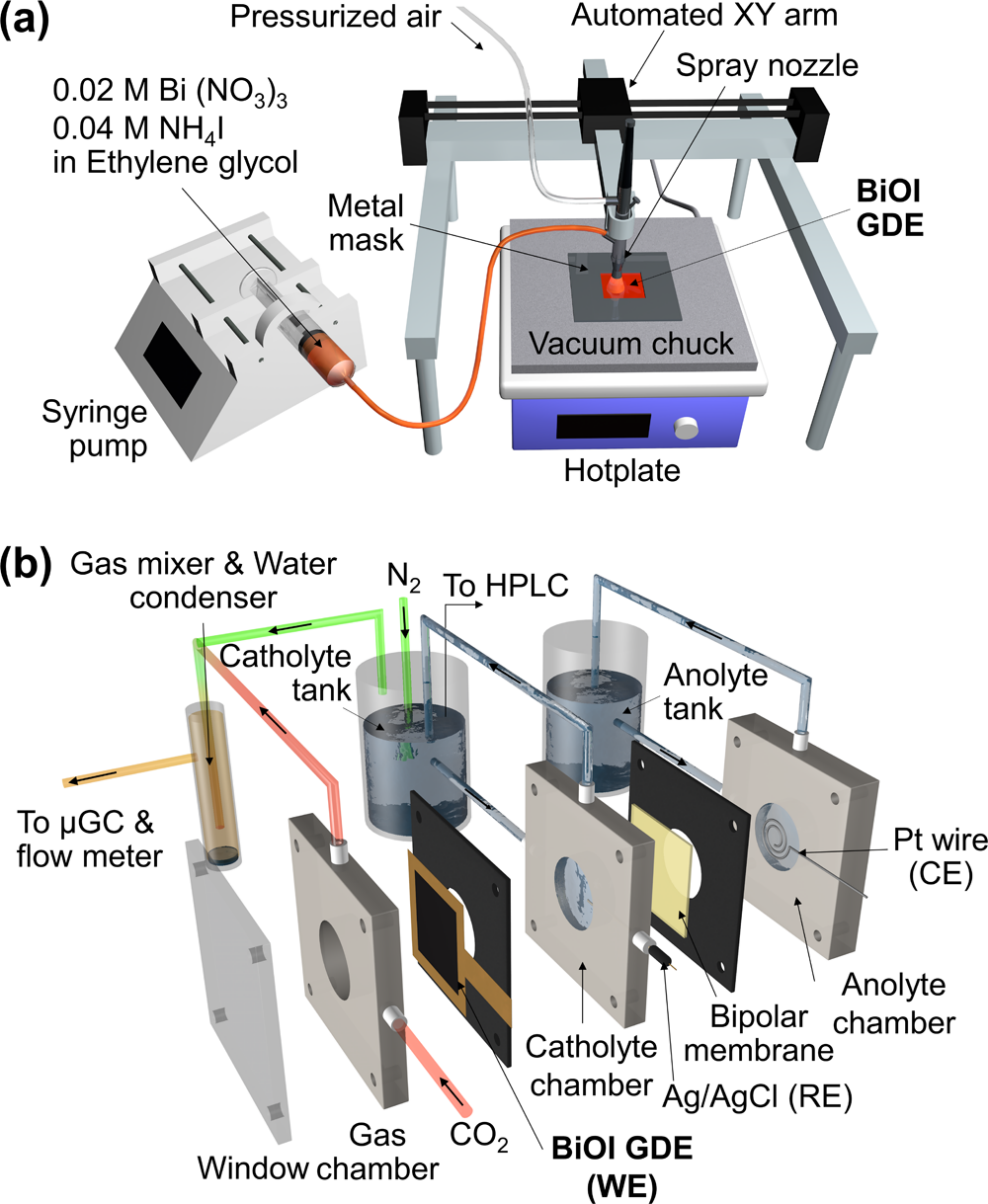
(a) Spray pyrolysis setup. (b) Schematic illustration of the CO_2_RR flow cell.

BiOI-coated GDEs were fabricated onto commercial gas diffusion paper (Ion Power, Sigracet 28BC) via spray pyrolysis at varying temperatures. First, BiOI precursor consisted of 0.02 M Bi(NO_3_)_3_ ·5H_2_O (98%, Carlo Erba) and 0.04 M NH_4_I (98%, Carlo Erba) in 30 mL of ethylene glycol (99.5%, Qrec) was freshly made. After thorough mixing, the precursor was loaded into a syringe pump to be fed into an automatic spray system, as shown in Figure 1a. The gas diffusion paper was mounted on a hot plate via a vacuum chuck and topped with a stainless-steel mask to control the deposition area to 3 × 3 cm^2^. The substrate was heated to achieve a surface temperature of 150, 200, and 250 °C as measured by a thermocouple, corresponding to the hot plate set point temperature of 250, 300, and 400 °C (maximum set point temperature), respectively. Then, the solution was dispensed at a rate of 0.5 mL min^–1^ while the spray nozzle moved line-by-line to cover the entire exposed area of the preheated substrate. The BiOI loading was controlled to 1.5 mg cm^–2^ by adjusting the spray volume.

The crystal structure of BiOI films prepared at different temperatures and under varying conditions was characterized by using grazing incident X-ray diffraction (GI-XRD, Rigaku TTRAX III) at an incident angle of 0.4°. Lattice parameter fitting was performed in FullProf software. The catalyst morphology was examined by using scanning electron microscopy (SEM, Hitachi SU8030) and transmission electron microscopy (TEM, JEOL JEM2100Plus, operated at 200 keV). The thickness and particle size were analyzed from the SEM images, and the lattice spacings were measured from TEM images via ImageJ software. The exposed crystal facet was identified by the selected area electron diffraction (SAED) technique in TEM. X-ray photoelectron spectroscopy (XPS, Shimadzu Kratos AXIS Supra+) was performed to determine the chemical states and compositions of the as-prepared BiOI electrodes. The binding energy calibration was done by setting the sp^3^ C 1s peak to 284.8 eV, and the spectra were deconvoluted in CasaXPS software.

### Electrochemical Experiment and Product Detection

The CO_2_RR experiments were conducted in a flow cell reactor as shown in Figure 1b. BiOI electrode served as the working electrode (WE) with an active surface area of 3.14 cm^2^. Pt wire (99.9%, Alfa Aesar) and leakless Ag/AgCl (eDAQ) were used as a counter electrode (CE) and a reference electrode (RE), respectively. The WE and CE were separated by a PEEK-reinforced bipolar membrane (BPM, Fumasep FBM-PK) to prevent the crossover of anion products. 1 M KOH (99.99% purity, Sigma-Aldrich) was used as an electrolyte. 40 mL of catholyte and anolyte were circulated between the reservoirs and the reaction chambers by peristaltic pumps at a flow rate of 20 mL min^–1^. Meanwhile, 99.995% of the CO_2_ gas was fed to the carbon side of the BiOI electrode at a constant flow rate of 30 mL min^–1^. When a potential was applied to the working electrode, the majority of the gas products remained in the gas chamber and were directed to a liquid condenser before composition analysis. However, there may be a small portion of the gas products that crossed over the catholyte side. Hence, 10 mL min^–1^ of 99.999% N_2_ stream was employed to purge out the dissolved gas products from the catholyte reservoir and the N_2_ purge line was combined with the main gas outlet. The combined gas products were analyzed using an online microgas chromatography (μGC, Varian CP4900) equipped with Molsieve 5A and PoraPLOT Q columns. Ar and He were used as reference gases for the thermal conductivity detector (TCD). The gas flow rate out of the reactor was measured using a film flow meter (Horiba SF-2U) and the real flow rate was used in the Faradaic efficiency calculation. The amounts of gas products were calculated using an average of at least 3 GC peak areas and volume flow rates, which may have resulted in modest overestimation (<5% FE) in some data points. Lastly, at the end of each electrolysis experiment, the liquid product was collected for analysis using high-performance liquid chromatography (HPLC, Shimazu) via a Shodex Sugar SH1011 Column.

All electrochemical experiments were collected using a PARSTAT MC potentiostat (AMETEK PMC-2000A). First, cyclic voltammetry (CV) measurements were performed from −0.9 to −2.0 V vs Ag/AgCl at a scan rate of 10 mV s^–1^ to activate the catalyst. The CO_2_RR was conducted under chronopotentiometry (CP) mode at 100, 150, 200, 250, and 300 mA cm^–2^ for 30 min per current without changing the sample. Prior to the CP experiment, electrochemical impedance spectroscopy (EIS) measurement at open-circuit potential was conducted at the frequency range of 100 kHz to 0.1 Hz and an amplitude of 10 mV. The resulting impedance value, an indicator of the solution resistance, was utilized for *iR*-compensation. The Ag/AgCl scale was converted to the reversible hydrogen electrode (RHE) using an experimentally calibrated value obtained with a Pt WE in a H_2_-purged electrolyte.

The Faradaic efficiency (FE) of the electrode was calculated using the following equation
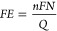
where *n* is the number of electrons required to obtain 1 molecule of the product. For CO, H_2_, and formate, 2 electrons are required. *N* is the moles of the product calculated from the molar concentration determined by GC or HPLC and the total gas or liquid volume, *Q* is the total charge passed recorded during electrolysis, and *F* is the Faraday constant (96,485 C mol^–1^).

The double-layer capacitance (*C*_dl_) was estimated from the nonfaradaic regions between −0.45 and −0.55 V vs Ag/AgCl at different scan rates ranging from 100 to 5 mV s^–1^. The *C*_dl_ was determined from the slope of the average cathodic and anodic current plotted against the scan rates. The stability of the BiOI electrode was examined at 100 mA cm^–2^ with a CO_2_ feed rate of 30 mL min^–1^, and the electrolyte was renewed every 12 h during the operations.

### In Situ and Ex Situ Spectroscopy

The BiOI electrode under CO_2_RR conditions was further analyzed by Raman spectroscopy (Horiba LabRAM HR Evolution) in a custom-made in situ Raman flow cell. CO_2_ gas reactant was continuously fed through the carbon side of the GDE at a constant flow rate of 10 mL min^–1^. Simultaneously, a 1 M KOH electrolyte was fed to the reactor at a flow rate of 10 mL min^–1^. Raman spectroscopy was performed using a 633 nm excitation laser with 50% power, 50× magnification objective lens, an acquisition time of 8 s and 8 accumulations, and 300 gr/mm grating. To examine intermediates in the CO_2_ reduction process for formate production, data were collected by applying a constant current density ranging from 10 to 80 mA cm^–2^.

As-prepared and spent BiOI electrodes were characterized by ex situ X-ray absorption near-edge structure (XANES) and extended X-ray absorption fine structure (EXAFS) conducted at beamline 1, Synchrotron Light Research Institute (SLRI), Nakhon Ratchasima, Thailand. The double-crystal monochromator (DCM) for the collimating beam was Ge(220). The measurements were performed in transmission mode for the Bi L3 edge. Data reductions were performed using Demeter programs including Arthena and Artemis. Bi foil, Bi_2_O_3_, and Bi_2_O_2_CO_3_ were used as standard materials. The composition of Bi species was performed using the linear combination fit (LCF) method on both XANES and EXAFS data. The goodness of fit was optimized to be higher than 95%.

## Results and Discussion

### Effects of Deposition Temperature on Morphology, Crystallinity, and Chemical Composition

In this spray pyrolysis method, BiOI is grown from a Bi(NO_3_)_3_·5H_2_O-NH_4_I precursor. Ethylene glycol serves as a solvent and stabilizer, creating an acidic environment that prevents premature hydrolysis of Bi(NO_3_)_3_ by moisture (eq 1). At high temperatures, ethylene glycol vaporizes, leaving Bi^3+^ and I^–^ to react in the air to form BiOI as shown in eq 2.^38^
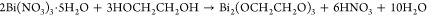
1
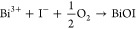
2

At high temperatures, all of the counterions undergo thermal decomposition and oxidation, leaving the substrate as vapor (eqs 3–5).
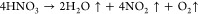
3
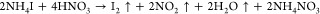
4
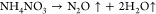
5

The substrate temperature affects not only crystal growth but also the rate of vaporization and oxidation of the solvent and counterions. Altering the substrate temperature influences the local environment during crystallization, which impacts the chemical and morphological properties of the catalysts.

**Figure 2:**
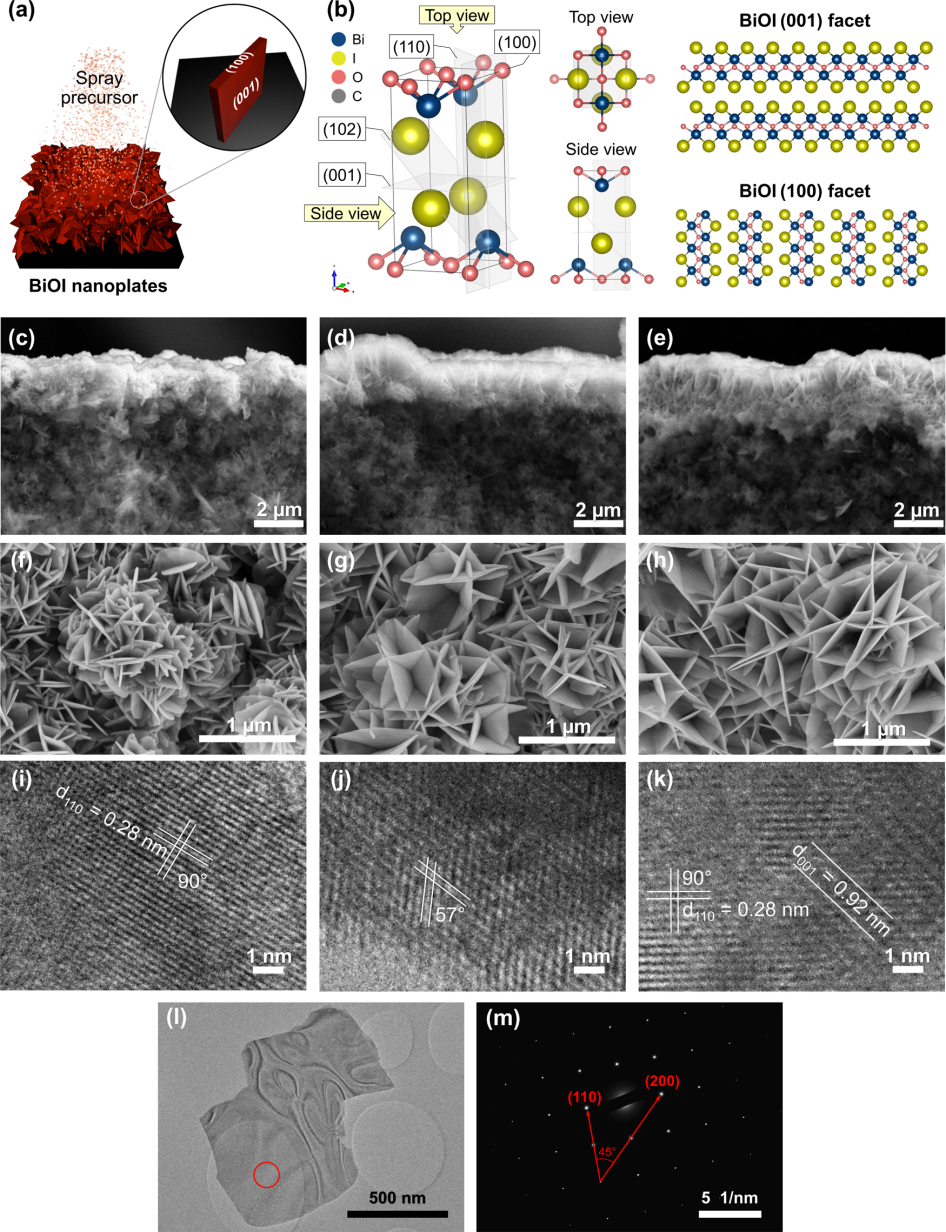
(a) Illustration of the as-deposited BiOI nanoplates synthesized by spray pyrolysis at high temperatures, showing the coexistence of (001) and (100), which are the major facets of the particles. (b) BiOI crystal structure and the atomic orientations of the (001) and (100) facets. (c–e) Cross-sectional backscattered electron images, (f–h) top-view SEM images, and (i–k) TEM images of the BiOI films deposited at 150, 200, and 250 °C, (l–m) SAED area, and SAED image of a BiOI nanoplate obtained from the 250 °C electrode, respectively.

The morphology of BiOI-150, BiOI-200, and BiOI-250 films was investigated using SEM. Although all BiOI samples exhibited a plate-like structure, variations in the deposition temperature significantly influenced the size, orientation, dimensions, and agglomeration tendencies of the BiOI crystals. Backscattered electron images in Figure 2c–e reveal that the films, which are lighter in color due to the substantially greater atomic weight of bismuth film compared to carbon substrate, had a median thickness of approximately 1.5, 1.6, and 1.7 μm for BiOI-150, BiOI-200, and BiOI-250, respectively (Figure S1a). As the deposition temperature increased, the BiOI plates broadened, transitioning from a median size of 225 nm in BiOI-150 to 475 nm in BiOI-250 (Figure S1b). Concurrently, the plate thickness slightly decreased from 52 to 28 nm, as depicted in Figure 2f–h (Figure S1c). In the case of BiOI-150, with a smaller plate size, the particles stacked randomly and agglomerated into large clusters. Conversely, for BiOI-250, the base of the nanoplates seemed to extend all the way to the carbon substrate, resulting in fewer grain boundaries and a more ordered crystal orientation perpendicular to the substrate. BiOI-200 exhibited a coexistence of both small and large crystal domains, similar to that of BiOI-150 and BiOI-250, respectively.

High resolution TEM (HRTEM) analysis shown in Figure 2i–k further elucidates the exposed facet orientations of the BiOI nanoplates. Most of the exposed facets show a lattice spacing of 0.28 nm with an angle of 90° between the two crystal planes (Figure 2i,k). These correspond to the angle between (110)/(110) planes as viewed from the (001) zone axis. The (012)/(212) planes as observed from the (100) zone axis with *d* spacings of 0.33 and 0.20 nm and an angle of 57° are also present as shown in Figure 2j.^39^ Prior studies postulated that the BiOI typically grows within the nonbonding layer of [I–Bi–O–Bi-I], perpendicular to the *c*-axis.^30^ Hence, it is likely that most of the exposed facet is the (001) plane, forming the faces of the plates, while the exposed width of the plates was mainly the (100) plane, as shown in Figure 2a. Hence, increasing deposition temperatures possibly induced growth of the (001) plane while inhibiting growth in the (100) plane. SAED was conducted on a relatively large particle (particle size >500 nm) with a selected area as shown in Figure 2l. Figure 2m reveals sharp spots, indicating that the particle is single crystalline. The spot pattern shows the angle of 45°, which is the theoretical value of the angle between the (110) and (200) planes of the tetragonal symmetry group of BiOI, thus indexing the diffraction spots along the [001] axis.^40^ This evidence confirms that the BiOI particles are mostly exposed with the (001) facet.^41^

**Figure 3:**
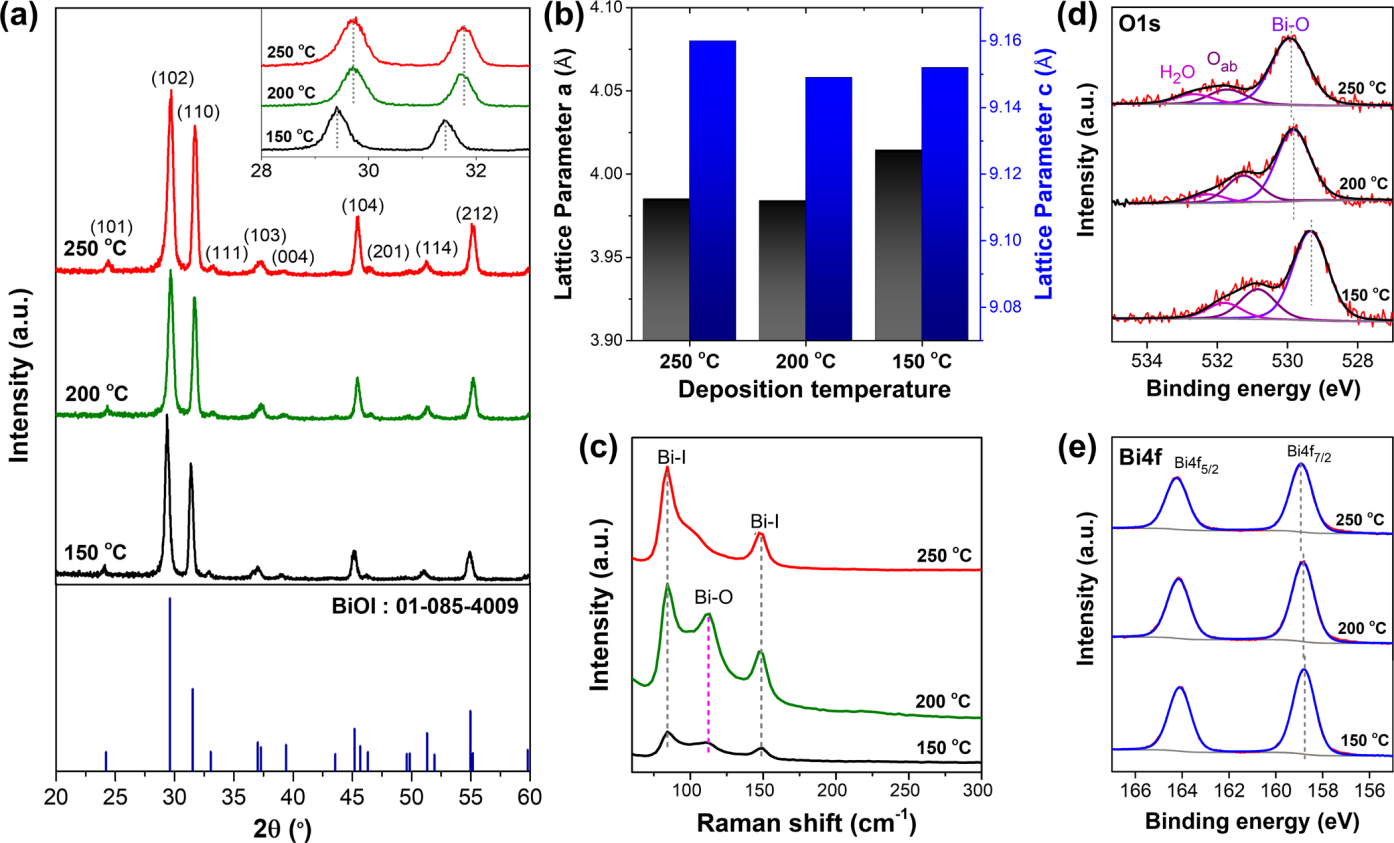
Physical and chemical properties of BiOI electrodes deposited at varying temperatures: (a) XRD patterns, (b) extracted lattice parameters, (c) Raman spectra, and XPS spectra of (d) O 1s and (e) Bi 4f.

The crystallographic structure of the BiOI electrodes, deposited at different temperatures, was investigated by using GI-XRD. Figure 3a depicts the XRD patterns of BiOI-150, BiOI-200, and BiOI-250. All electrodes exhibited a diffraction pattern consistent with the tetragonal phase of BiOI (JCPDS: 085–4009), with a preferred orientation along the (102) and (110) planes. The intensity ratio between the (110) and (102) planes increased with the deposition temperature, measuring values of 0.70, 0.80, and 0.82 for BiOI-150, BiOI-200, and BiOI-250, respectively. This trend corresponds well with the augmented growth of the (001) facet observed in the SEM images. Furthermore, BiOI-150 exhibited a noticeable peak shift toward lower angles, as shown in the inset of Figure 3a. Lattice parameter fitting unveiled that BiOI-150 exhibited a 0.7% expansion in lattice parameter a and a 0.1% reduction in parameter c compared to BiOI-250, resulting in a 0.7% increase in the unit cell volume (Figure 3b and Table S2). This expansion indicates distortion in the BiOI crystal structure, consistent with reported iodide-deficient BiOI structures.^30,42^

Ex situ Raman spectra of all three electrodes as depicted in Figure 3c further supported the XRD results. Specifically, BiOI-150 and BiOI-200 exhibited a prominent peak at 112.14 cm^–1^, corresponding to the Bi–O bond in the β-Bi_2_O_3_ phase.^43^ This Bi–O peak was notably absent in the case of the BiOI-250. This observation suggested that BiOI crystals synthesized at lower temperatures contained a significant amount of bismuth oxide. Surface analysis by XPS, as shown in Table 1, also confirmed that the electrodes prepared at lower temperatures contained a higher amount of oxygen and a lower amount of iodide within the structure.^30^ The presence of excess oxygen in BiOI-150 was also evident in the O 1s spectra shown in Figure 3d, where a significant fraction of adsorbed oxygen (O_ad_) was detected. Notable shifts to lower binding energy were also found in the Bi 4f spectra of BiOI-150 shown in Figure 3e, which could be attributed to the presence of iodide vacancies or the higher oxygen concentration surrounding both the Bi and I elements, as demonstrated in Table 1 and Figure S2.^44^

**Table 1:** Atomic Compositions of Bi, O, and I Elements in BiOI-150, BiOI-200, and BiOI-250 Electrodes, as Measured by XPS

	atom %			
deposition temperature (°C)	Bi	O	I	Bi/I
250	27	21	27	1.0
200	28	22	24	1.2
150	27	24	19	1.4

All ex situ characterizations suggest that the spray pyrolysis temperature significantly affects the particle size, facet dimension, crystal orientation, and phase purity. Specifically, higher temperatures promote the growth of the (001) plane (inhibit the [001] direction), induce crystal orientation perpendicular to the substrates, and reduce phase impurities due to iodide vacancies. As shown in the synthesis scheme (eqs 1–5), spray pyrolysis temperature directly impacts not only the growth rate of BiOI nanoplates but also the chemical environment during crystallization. At low temperatures, the solvent, byproducts, and counterions may not fully react and vaporize, leading to nonideal local environments. It has been shown that an increased pH can induce partial substitution of I^–^ in BiOI by OH^–^,^30^ resulting in the loss of iodide and a substantial expansion in the unit cell. These factors can influence the CO_2_RR performance.

### Effects of Deposition Temperature on CO_2_RR Activity and Stability

**Figure 4:**
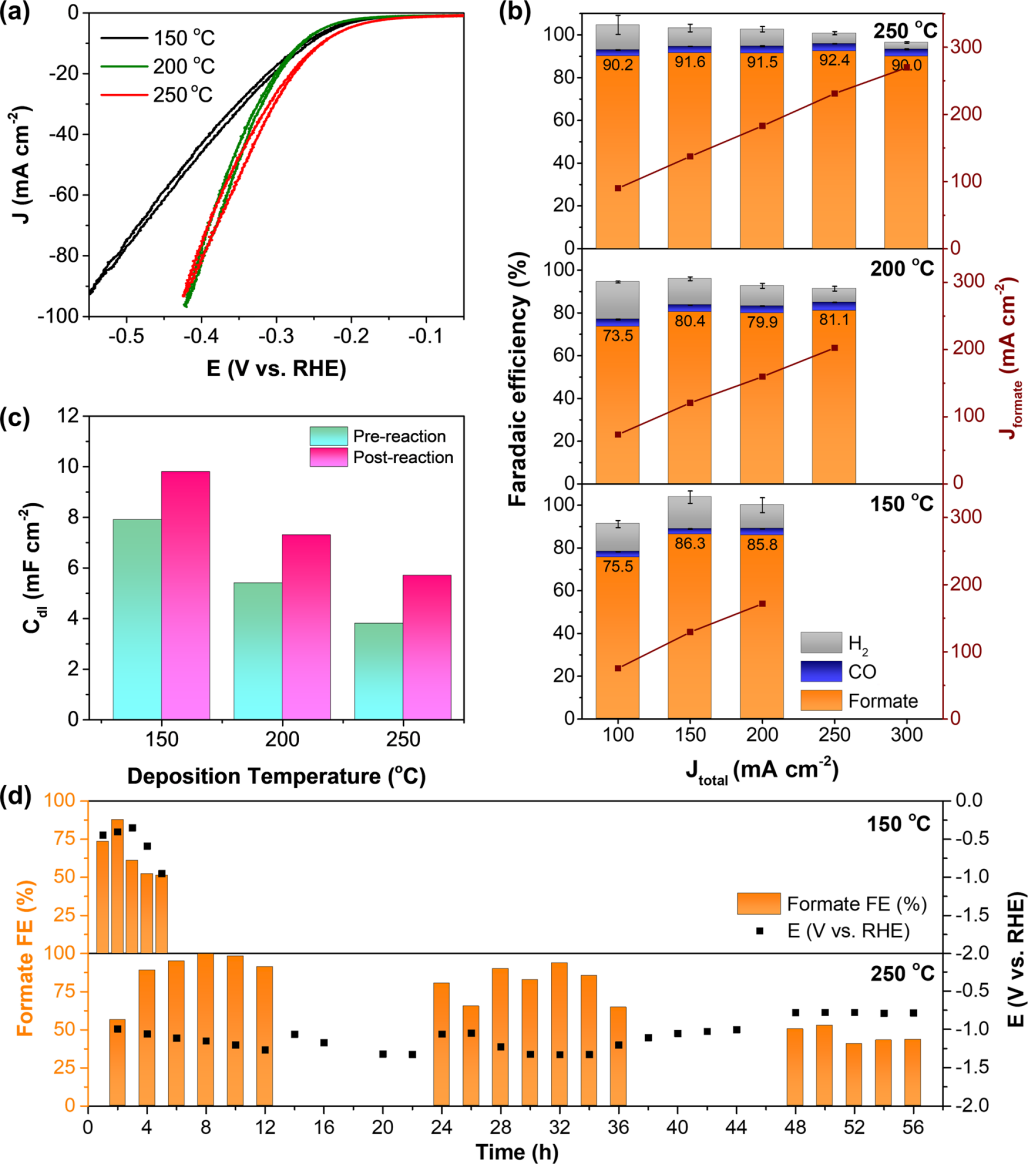
(a) Cyclic voltammetry of BiOI GDEs tested in a CO_2_RR flow cell using 1 M KOH electrolyte. (b) Faradaic efficiency of BiOI GDEs with increasing current densities and (c) double-layer capacitances of BiOI electrodes. (d) Stability test of BiOI-150 and BiOI-250 at 100 mA cm^–2^.

The CO_2_RR performance of BiOI-150, BiOI-200, and BiOI-250 was assessed in a flow cell reactor by using 1 M KOH as the electrolyte. Initially, each electrode underwent an activation process via cyclic voltammetry (CV) to achieve a stable phase. The stable CV scan, depicted in Figure 4a, revealed that BiOI-250 displayed a slightly earlier onset potential, surpassing the other two electrodes by at least 30 mV. Particularly, the CV of BiOI-150 exhibits a markedly linear current–voltage curve even after compensating for solution resistance, suggesting that the sample may experience higher intrinsic resistance than BiOI samples synthesized at higher temperatures. The resistive nature of BiOI-150 may originate from its small, heavily defected, and randomly oriented nanoplates.

To evaluate formate selectivity, chronopotentiometry experiments were conducted on the same electrodes, subjecting it to increasing current densities from 100 to 300 mA cm^–2^ for 30 min per current or until the electrode could no longer perform CO_2_RR. Gas products, namely, H_2_ and CO, were continuously monitored using online μGC throughout the experiment. Additionally, after electrolysis, the liquid products were quantified by HPLC. The CO_2_RR performances of the BiOI electrodes are presented in Figure 4c, revealing that formate was the main product, with minor amounts of H_2_ and CO detected. BiOI-150 and BiOI-200 exhibited higher Faradaic efficiency (FE) of H_2_, ranging between 6 and 18% compared to that of BiOI-250 (3–11%). However, the CO FE of all electrodes was at approximately 2–3%. When considering just the Faradaic efficiency of formate and CO, we found that all electrodes exhibited a similar formate selectivity of around 96–97% (Figure S3), suggesting that the CO_2_RR active sites on all electrodes were similar. In terms of formate yield, the highest achievable partial current density for formate was 170 mA cm^–2^ for BiOI-150, 202 mA cm^–2^ for BiOI-200, and 270 mA cm^–2^ for BiOI-250. This superior activity arises from BiOI-250’s ability to withstand higher current densities, up to 300 mA cm^–2^, without flooding.

Previously suggested causes of flooding in GDE systems include factors such as electrowetting, water pumping, salt precipitation, pressure differentials between gas and liquid interfaces, and catalyst restructuring under high potential.^45−47^ To understand the cause of flooding in our system, the double-layer capacitances (*C*_dl_) of BiOI electrodes before and after the chronopotentiometry experiments were determined. As shown in Figures 4d and S4, the *C*_dl_ values were 7.9, 5.4, and 3.8 mF cm^–2^ for CV-activated BiOI-150, BiOI-200, and BiOI-250, respectively. These *C*_dl_ values correlate well with the SEM images (Figure 2f–h), where smaller particle sizes correspond to higher *C*_dl_ values. Following the reaction at 100 mA cm^–2^ for 30 min, the *C*_dl_ of all electrodes increased. Typically, for electrocatalysts, a large *C*_dl_ is preferable, as it indicates a higher electrochemically active surface area (ECSA) and more reaction sites. However, in the case of gas diffusion electrodes, a higher *C*_dl_ implies greater exposure to the electrolyte, which may lead to a higher tendency for flooding. The increase in *C*_dl_ after testing suggested that the electrowetting effect may be one of the failure mechanisms of the BiOI electrodes.

The long-term stability of BiOI-150 and BiOI-250 was further assessed at a constant current density of 100 mA cm^–2^. The formate FE of BiOI-150 declined to only 50% after 4 h of operation, while that of BiOI-250 maintained above 90% for over 34 h, as depicted in Figure 4e. The BiOI-150 electrode exhibited a significant potential increase after 4 h, leading to the generation of H_2_ and subsequent electrode flooding. In contrast, BiOI-250 maintained a stable potential within the range of −0.9 to −1.3 V vs RHE, showcasing its capacity to efficiently generate formate at high FE for the first 34 h and at a moderate FE without flooding for an additional 22 h. This proved that BiOI-250 remained functioning for a much longer time than the BiOI-150, which perished tragically as a result of macroscopic flooding. These results illustrated that both the activity and stability of the electrode are intricately linked to the morphology and phase of the BiOI catalysts.

### Elucidating the Activity Trends with In Situ Raman Spectroscopy and Ex Situ X-ray Absorption Spectroscopy

**Figure 5:**
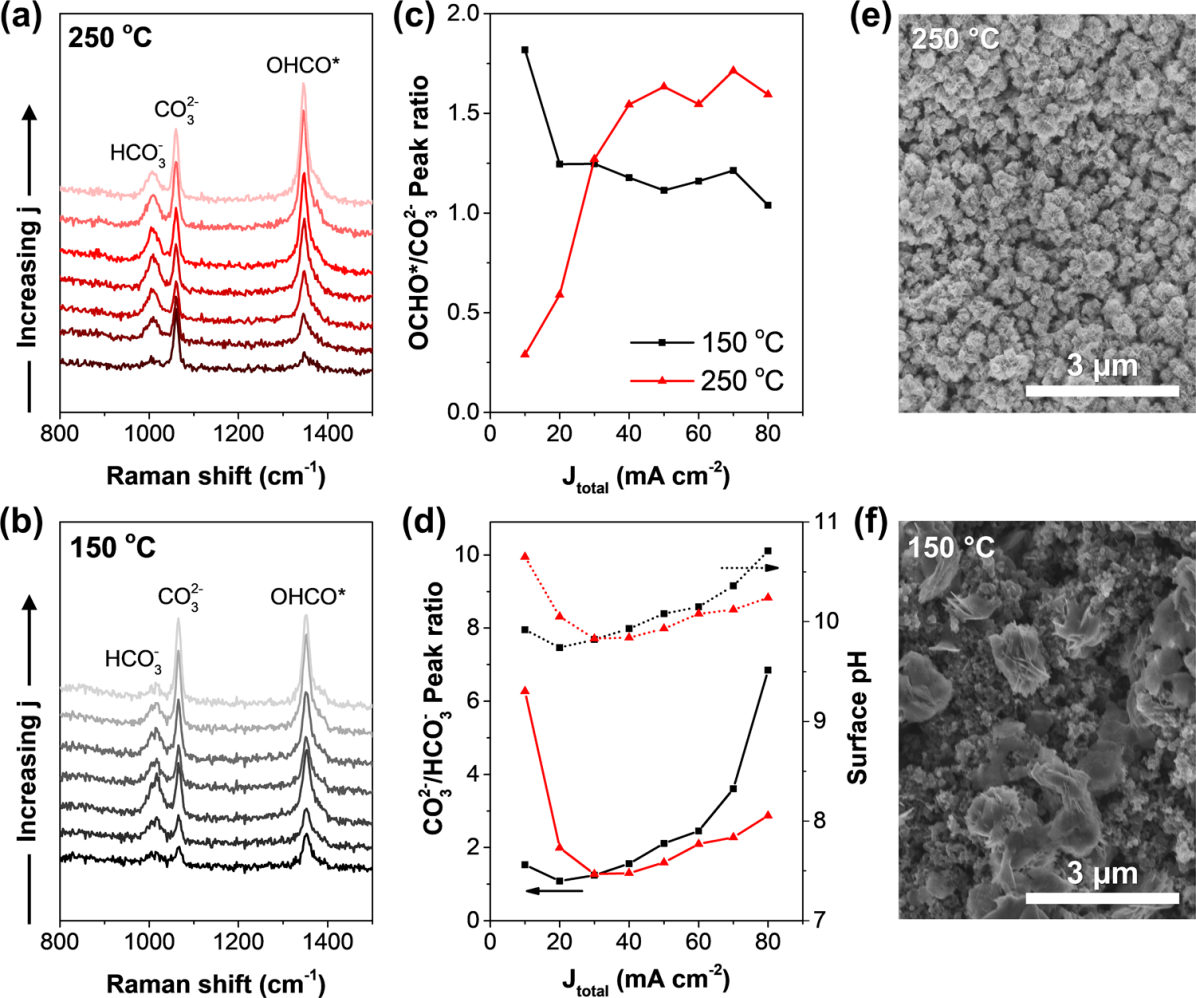
In situ Raman spectra of BiOI electrodes deposited at (a) 250 °C and (b) 150 °C collected under CO_2_RR conditions at varying current densities from 10–80 mA cm^–2^. (c) Ratio of *OCHO/CO_3_^2–^ peak intensity as a function of current and (d) ratio of the CO_3_^2–^/HCO_3_^–^ peak intensity and the corresponding surface pH derived from the Bjerrum plot of carbonate equilibria. SEM images of the spent (e) BiOI-250 and (f) BiOI-150 after 100 mA cm^–2^ for 30 min.

To delve deeper into the CO_2_RR activity trend of BiOI deposited at various temperatures, we employed in situ Raman spectroscopy. The CV-activated BiOI-150 and BiOI-250 were subjected to chronopotentiometry experiments with increasing current densities from 10 to 80 mA cm^–2^ and the structural and chemical changes were characterized by a confocal Raman microscope. As shown in Figure 5a,b, under CO_2_RR conditions, both samples exhibited Raman shift at 1348–1350 cm^–1^, corresponding to the *OCHO intermediate. This observation suggests that the CO_2_RR pathway on both electrodes similarly proceeded through the *OCHO intermediate.^48^ Moreover, peaks corresponding to CO_3_^2–^ surface adsorption at 1060–1065 cm^–1^ and HCO_3_^–^ adsorption at 1011–1014 cm^–1^ were detected under CO_2_RR conditions. These anions formed as CO_2_ reacted with OH^–^ from the electrolyte and electrolysis byproducts, establishing a pH-dependent equilibrium among OH^–^, HCO_3_^–^, and CO_3_^2–^ species, as described by the Bjerrum plot of carbonate equilibria (Figure S5).^49^ Comparing the intensities of HCO_3_^–^ and CO_3_^2–^ can provide insights into the local pH of the electrode surface.^50^ Increasing the applied current density on these electrodes led to not only higher *OCHO peak intensities but also elevated CO_3_^2–^ peak intensities, indicating an increase in the rates of CO_2_RR and carbonation. The latter is attributed to the higher OH^–^ concentration produced through electrolysis, which increased the surface pH, elevating the CO_3_^2–^ concentration relative to that of HCO_3_^–^.

Notably, normalizing the *OCHO peak intensity against the CO_3_^2–^ peak intensity revealed a significant difference in the coverage of the intermediates on both electrodes. As illustrated in Figure 5c, the *OCHO/CO_3_^2–^ intensity ratio appeared to increase with the current density for BiOI-250, whereas the reverse trend was observed for BiOI-150. Furthermore, the CO_3_^2–^/HCO_3_^–^ peak ratio for BiOI-250 seemed to increase linearly with the current density, while an exponential increase was noted in the case of BiOI-150, particularly when the current exceeded 60 mA cm^–2^. This observation indicates that BiOI-250 effectively maintained the equilibrium between CO_2_RR and carbonation, leading to a gradual change in surface pH (Figure 5d). In contrast, the surface of BiOI-150 became progressively more alkaline, with the rate of carbonation surpassing that of the CO_2_RR. These findings may be attributed to microscale flooding of the catalyst layer, leading to an excess of OH^–^ produced from the HER, resulting in more pronounced changes in the CO_3_^2–^ coverage compared to *OCHO coverage with increasing current. The increase in local pH correlated with the rise in reduction potential, notably observed as BiOI-150 began to flood at the fourth-hour mark during stability testing (Figure 4e). This heightened flooding propensity serves as a precursor to the inferior CO_2_RR selectivity and macroscopic instability of BiOI-150 compared to BiOI-250.

Further insights from SEM analysis revealed that after the CO_2_RR, the structure of BiOI-250 remained intact (Figures 5e and S6), whereas that of BiOI-150 underwent substantial restructuring and visible collapse, particularly in areas near the substrate (Figure 5f). This indicates that a critical failure mechanism for BiOI-150 is catalyst restructuring, resulting in significantly enhanced wetting and a reduced rate of the CO_2_RR with increasing current density. These results underscore the crucial role of BiOI nanoplate morphology in maintaining a high CO_2_RR activity and stability. With smaller, highly defective, and randomly oriented nanoplates, BiOI-150 was likely significantly less conductive, as electrons had to traverse many grain boundaries and across BiOI sheets instead of primarily in-plane conductivity as in the case of BiOI-250. This is evident in the operating potential under the same current density. At 200 mA cm^–2^, the BiOI-150 exhibited a significantly higher potential at −0.54 V vs RHE compared to −0.43 V vs RHE for BiOI-250. The high operating potential may be a result of nonuniform potential distribution, where conductive areas near the carbon substrates experience higher reduction potentials, while less conductive clusters on top of the electrode remain relatively inactive.^51^ This led to increased HER and ultimately flooding, thereby slowing down the rate of CO_2_RR due to an extended CO_2_ diffusion path length.^45,52^ Eventually, the BiOI structures near the carbon substrate collapsed, resulting in a catastrophic failure.

**Figure 6:**
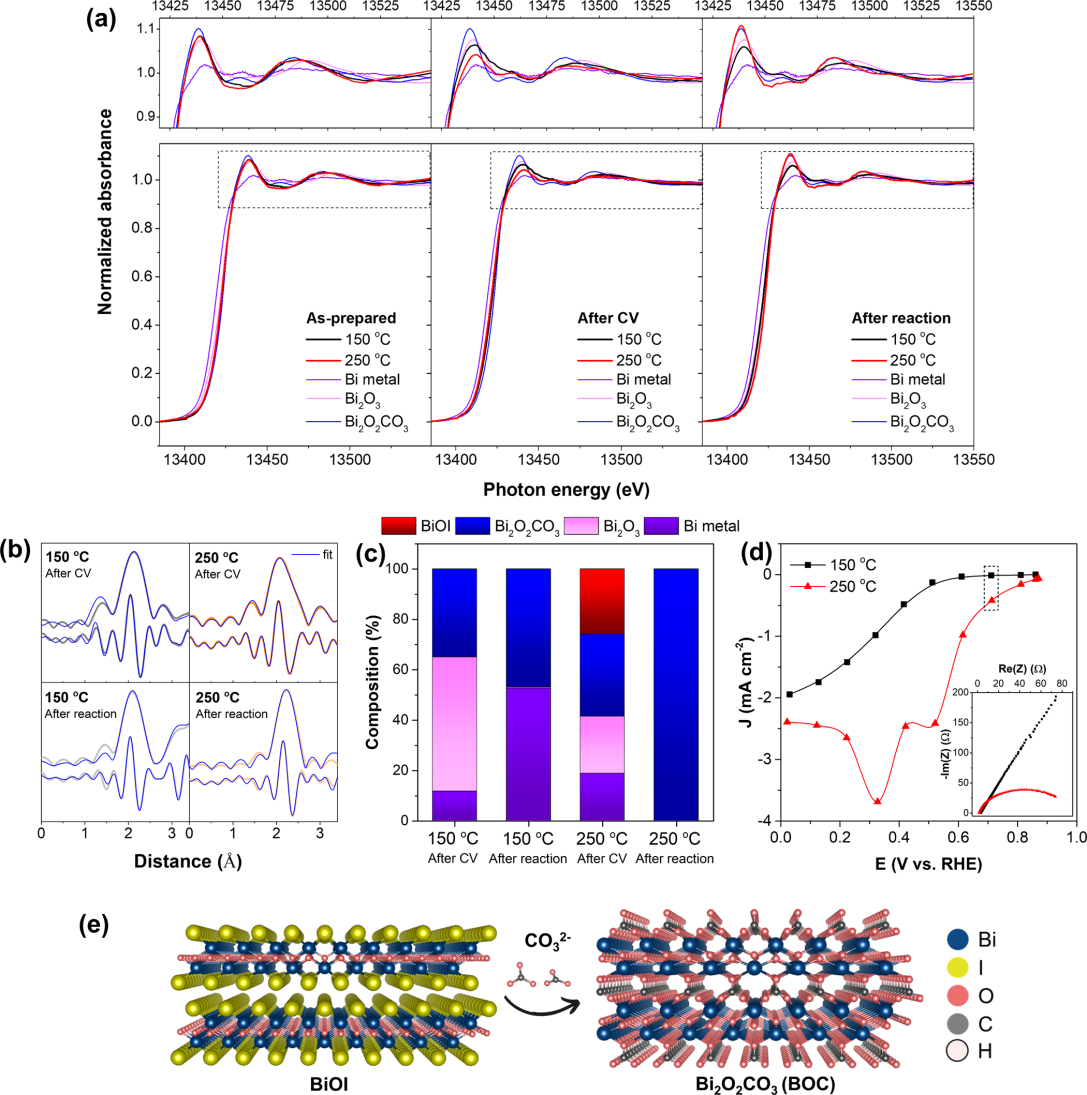
(a) XANES and (b) FT-EXAFS spectra at Bi L3 edge for BiOI electrodes at varying conditions. (c) Electrode compositions obtained from linear combination fit of XANES and EXAFS spectra, with higher than 95% goodness of fit. (d) Reduction peaks of fresh BiOI electrodes and the impedance spectra collected at 0.71 V vs RHE. (e) Structural transformation of BiOI to the Bi_2_O_2_CO_3_ active phase.

One lingering question is why BiOI-150 exhibited higher resistance compared to that of BiOI-250. This question was addressed by investigating the phase changes in a BiOI electrode during CO_2_RR using X-ray absorption spectroscopy (XAS). BiOI-150 and BiOI-250, under varying conditions from freshly deposited to CV-activated to postreaction under 100 mA cm^–2^ for 30 min, were characterized using X-ray absorption near-edge structure (XANES) and extended X-ray absorption fine structure (EXAFS), with the results presented in Figure 6a,b, respectively. By performing a linear combination fit (LCF) of both XANES and EXAFS (Tables S3–S4) using reference materials including Bi_2_O_3_, Bi_2_O_2_CO_3_, BiOI, and Bi metal, the compositions of the electrodes under different operating conditions were quantified. As depicted in Figure 6c, it was discovered that the CV-activated BiOI-150 consisted mainly of the Bi_2_O_3_ phase, which then transformed into a 1:1 mixture of Bi_2_O_2_CO_3_ and Bi metal after reduction. In contrast, BiOI-250, after CV activation, contained much less Bi_2_O_3_ and fully converted to pure bismuth subcarbonate (Bi_2_O_2_CO_3_) after the reaction. This finding aligns well with the XRD analysis presented in Figure S7a,b. The XRD patterns of the reduced BiOI-150 sample prominently display a Bi metal peak. In contrast, the XRD patterns of the reduced BiOI-250 samples, even after reduction at 300 mA cm^–2^, primarily exhibit peaks corresponding to Bi_2_O_2_CO_3_, as shown in Figure S8.

It has been proposed that the conversion of the CO_2_RR to formate on a BiOI catalyst may occur via two distinct active sites: Bi and Bi_2_O_2_CO_3_,^53^ with the latter offering superior activity and higher stability.^52,54^ The formation of Bi_2_O_2_CO_3_ involves the ion exchange of iodide ions with carbonate ions between (Bi_2_O_2_)^2+^ layers during the CO_2_RR (Figure 6e). This process was experimentally proven to be challenging when starting from the Bi_2_O_3_ phase as the CO_2_ insertion into Bi_2_O_3_ is less favorable than oxide reduction to metallic Bi, leading to a prominent Bi metal XRD peak found in the Bi_2_O_3_ electrode after CO_2_RR was conducted at 100 mA cm^–2^ (Figure S7c). The Bi metal phase that emerges during the reaction may hamper the efficiency of the CO_2_RR as it favors HER.^52^ As indicated by the XAS results, it is likely that the Bi_2_O_3_ impurity phase observed in XRD, XPS, and ex situ Raman spectroscopy was responsible for hindering the successful formation of the Bi_2_O_2_CO_3_ active phase. Further examination of the reduction potential of fresh BiOI electrodes revealed that while BiOI-250 showed an early reduction onset potential at 0.85 V vs RHE, BiOI-150 exhibited a much later reduction onset by at least 350 mV and significantly higher impedance (Figures 6d and S9). This confirms that the resistive Bi_2_O_3_ domains interfered with the transformation of BiOI into the desired Bi_2_O_2_CO_3_ phase.

Through in situ and ex situ characterizations, we elucidated how the crystallographic properties of BiOI synthesized via a straightforward spray pyrolysis technique at different temperatures influenced the electrode’s activity and stability. Our discovery showcases how the impurity phase in the BiOI crystal structure interferes with the structural formation of the Bi_2_O_2_CO_3_ active site, leading to high resistivity and catalyst restructuring. Consequently, microscopic flooding occurred, which macroscopically affected the overall selectivity and long-term stability of the catalyst.

## Conclusions

We successfully synthesized a highly active BiOI gas diffusion electrode with a nanoplate structure by using the single-step spray pyrolysis method. Variations in the electrode deposition temperature influenced several aspects of this nanoplate morphology, including width, plate orientation, exposed facet dimensions, and impurity phases. Under optimal deposition conditions at 250 °C, the pure-phase BiOI crystals exhibited growth along the [100] direction, exposing the (001) facet, which was oriented perpendicular to the substrate, leading to high electron conductivity. The BiOI-250 demonstrated superior formate faradic efficiency and the ability to withstand high currents without catastrophic flooding. It achieved up to 270 mA cm^–2^ formate partial current density and stability up to 34 h at 100 mA cm^–2^. In situ Raman spectroscopy unveiled that unlike the electrode synthesized at a lower temperature, BiOI-250 exhibited an increase in *OCHO coverage relative to CO_3_^2–^ with rising current density, indicating the electrode’s ability to effectively manage the rates of CO_2_RR and carbonation, thereby ensuring stable performance without macroscopic flooding. Ex situ XAS results unveiled that BiOI-250 fully converted to the active Bi_2_O_2_CO_3_ phase during the CO_2_RR. However, BiOI-150, which contained domains of resistive Bi_2_O_3_ impurity phase, formed a mixture of Bi_2_O_2_CO_3_ and less reactive metallic Bi. This impurity phase is the root cause of flooding in our system, contributing to the resistive pathway causing uneven potential distribution, resulting in structural collapse at high current density. Our discovery sheds light on material design criteria that may reduce the flooding tendency of gas diffusion electrodes for CO_2_RR in the future.

## Supporting Information Available

Survey of state-of-the-art Bi-based catalysts for formate, statistical analysis of SEM images, lattice parameters, I 3d XPS, formate selectivity, capacitive current density, pH calculation based on carbonate equilibrium, additional SEM images and XRD patterns, Nyquist plot, and XAS fitting results and parameters (PDF)
