# Supplementary material for: Single-Step Fabrication of BiOI Nanoplates as Gas Diffusion Electrodes for CO2 Electroreduction to Formate: Effects of Spray Pyrolysis Temperature on Activity and Flooding Propensity
Source: ACS Appl Nano Mater. 2024 Jul 24;7(17):20046–57. doi: 10.1021/acsanm.4c02570 (PMC11406483; doi:10.1021/acsanm.4c02570)
Supplement: Supplementary file 1 — an4c02570_si_001.pdf [file an4c02570_si_001.pdf]

Supporting Information

Single-Step Fabrication of BiOI Nanoplates as Gas Diffusion Electrodes for CO_2 Electroreduction to Formate: Effects of Spray Pyrolysis Temperature on Activity and Flooding Propensity

Kornkamon Meesombad[1], Kasempong Srisawad[1], Pongtanawat Khemthong[1], Teera Butburee[1], Chattarika Sukpattanacharoen[2], Kajornsak Faungnawakij[1]*, Pongkarn Chakthranont[1]*

[1] National Nanotechnology Center (NANOTEC), National Science and Technology Development Agency (NSTDA), Pathum Thani, Thailand, 12120

[2] Division of Innovation and Research, Department of Disease Control, Ministry of Public Health, Nonthaburi, Thailand, 11000

**Corresponding E-mail:* pongkarn.cha@nanotec.or.th, kajornsak@nanotec.or.th

Table S1. Activities of state-of-the-art Bi-based CO_2RR electrocatalysts for formate production reported in literature.

Catalyst	Electrolyte	FE formate (%)	$J_{formate}$ (mA cm^{-2})	Potential (V vs. RHE)	Stability (h)	Ref.
Bi_2S_3	1M KOH	93	1900	-0.90	100	26
Bi@ $Bi_2O_2CO_3$	1M KOH	100	1200	-0.70	16	27
Edge/defect-rich Bi nanosheets	1M CsOH	>90	870	-1.80	10	21
BiOON on PTFE	1M KOH	83	677	-0.70	6	7
Bismuthene (Bi-ene-NW)	1M KOH	91	570	-0.87	500	19
$Bi_2O_2CO_3$	1M KOH	100	300	-0.60	12	25
Bi_2O_3	1M KOH	98	288	-0.61	11	8
**Spray pyrolysis BiOI-250**	1M KOH	**>90**	**270**	**-1.0**	**34**	**This work**
$Bi_2O_2CO_3$	1M KOH	95.3	207.1	-1.2	-	28
Bi/GDE	1M KOH	93	200	-0.61	-	5
Bi_2O_3	1M KOH	93	200	-0.28	10	9
Bi-based MOF (CAU-17)	1M KOH	90	200	-0.52	>10	15
Bi_2S_3-Bi_2O_3 nanosheets	1M KOH	93.8	200	-1.10	18	14
$Bi_2O_2SO_4$	1M KOH	93.55	158.1	-1.10	16	24
Bi/GDE	1M KOH	92	155	-	10	6
BiOBr	1M $KHCO_3$	>90	148	-1.05	8	11
Bi_2O_3 NPs	0.5M $KHCO_3$	93.7	102.1	-1.7	12	10
Bi_2O_3	1M KOH	>70	85	-1.15	100	48
BiOI	0.5M $KHCO_3$	95.9	5.0	-0.77	16	12
BiOCl	0.5M K_2SO_4	86.2	8	-1	24	41

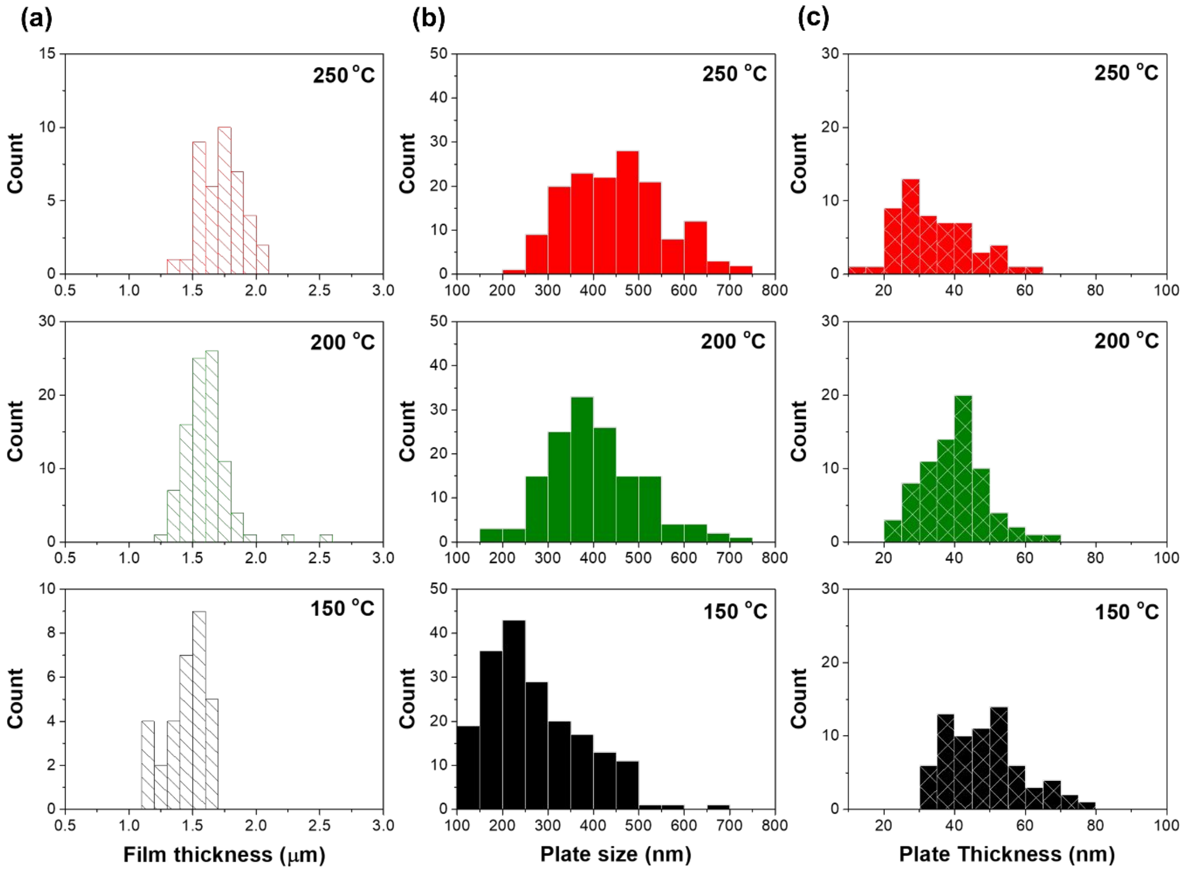

Figure S1. Statistical analysis of (a) film thickness, (b) nanoplate size, and (c) nanoplate thickness of BiOI electrodes deposited at varying temperatures of 150, 200, and 250 °C as measured in SEM images.

Table S2. Lattice parameters obtained from XRD patterns via Rietveld refinement

Synthesis temperature (°C)	Lattice parameter (Å)			Unit cell volume (Å^3)
	a	b	c	
**250**	3.985	3.985	9.160	146.40
**200**	3.984	3.984	9.149	145.88
**150**	4.014	4.014	9.152	147.49

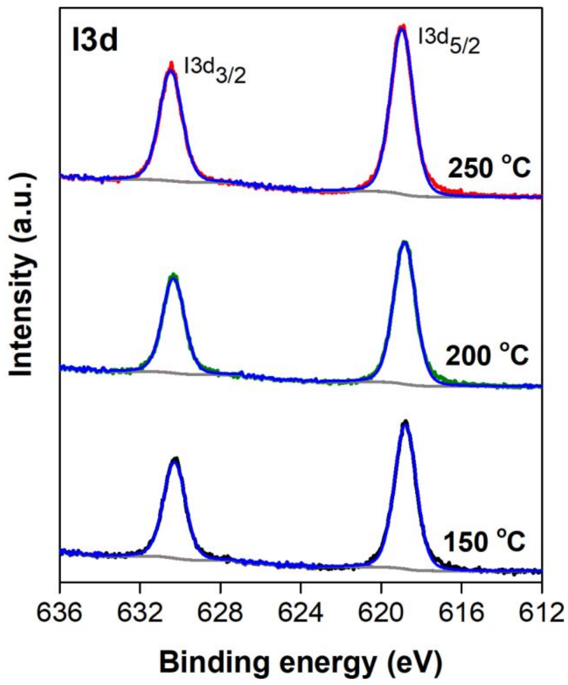

Figure S2. High-resolution XPS spectra of I3d of BiOI deposited at 150, 200, and 250 °C.

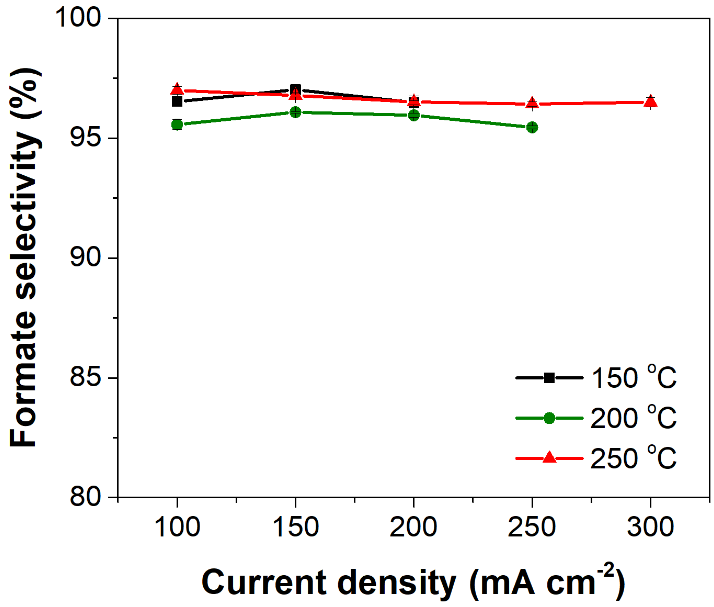

Figure S3. CO_2RR selectivity to formate (formate/(formate + CO)) of all electrodes as a function of total current density.

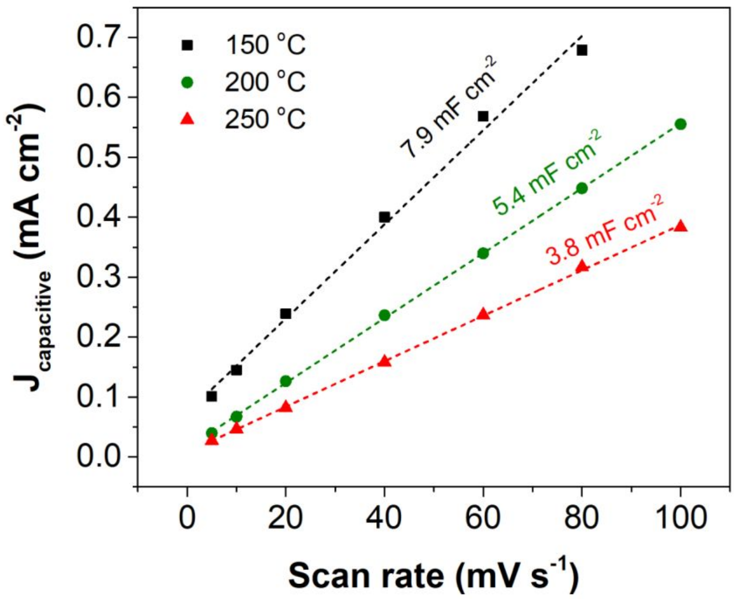

Figure S4. Capacitive current densities of BiOI electrodes obtained from the non-Faradaic region between -0.45 and -0.55 V vs. Ag/AgCl at different scan rates.

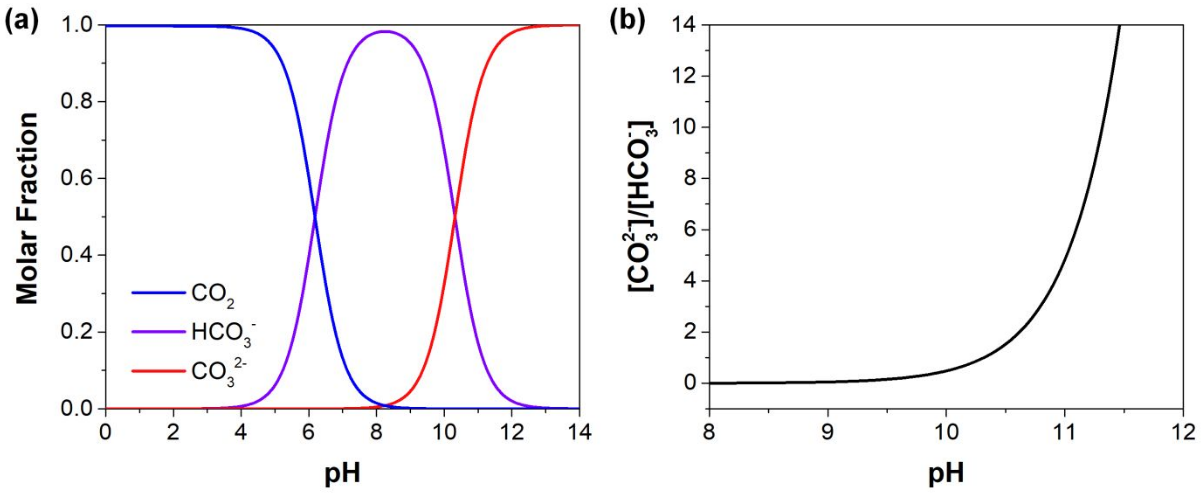

Figure S5. (a) Molar fraction of CO_2, HCO_3^-, and CO_3^{2-} in equilibrium and (b) the ratio of CO_3^{2-}/HCO_3^- as a function of pH.

Equilibrium equations

$$\mathrm{CO_2(aq) + H_2O \leftrightarrow H_2CO_3(aq)} \qquad \mathrm{K_d} = 2.63 \times 10^{-3}$$

$$\mathrm{H_2CO_3(aq) \leftrightarrow HCO_3^-(aq) + H^+(aq)} \qquad K_1 = 2.5 \times 10^{-4}$$

$$\mathrm{HCO_3^-(aq) \leftrightarrow CO_3^{2-}(aq) + H^+(aq)} \qquad K_2 = 4.84 \times 10^{-11}$$

$$\mathrm{K_d} = \frac{[\mathrm{H_2CO_3}]}{[\mathrm{CO_2}]},\ \mathrm{K_1} = \frac{[\mathrm{HCO_3^-}][\mathrm{H^+}]}{[\mathrm{H_2CO_3}]},\ \mathrm{K_2} = \frac{[\mathrm{CO_3^{2-}}][\mathrm{H^+}]}{[\mathrm{HCO_3^-}]}$$

$$Total = [\mathrm{CO_2}] + [\mathrm{H_2CO_3}] + [\mathrm{HCO_3^-}] + [\mathrm{CO_3^{2-}}]$$

$$\frac{[\mathrm{H_2CO_3}]}{Total} = \frac{1}{\frac{1}{\mathrm{K_d}} + 1 + \frac{\mathrm{K_1}}{[\mathrm{H^+}]} + \frac{\mathrm{K_1K_2}}{[\mathrm{H^+}]^2}}$$

$$\frac{[\mathrm{CO_2}]}{Total} = \frac{1}{1 + \mathrm{K_d} + \frac{\mathrm{K_1K_d}}{[\mathrm{H^+}]} + \frac{\mathrm{K_1K_2K_d}}{[\mathrm{H^+}]^2}}$$

$$\frac{[\mathrm{HCO_3^-}]}{Total} = \frac{1}{\frac{[\mathrm{H^+}]}{\mathrm{K_1K_d}} + \frac{[\mathrm{H^+}]}{\mathrm{K_1}} + 1 + \frac{\mathrm{K_2}}{[\mathrm{H^+}]}}$$

$$\frac{[\mathrm{CO_3^{2-}}]}{Total} = \frac{1}{\frac{[\mathrm{H^+}]^2}{\mathrm{K_dK_1K_2}} + \frac{[\mathrm{H^+}]^2}{\mathrm{K_1K_2}} + \frac{[\mathrm{H^+}]}{\mathrm{K_2}} + 1}$$

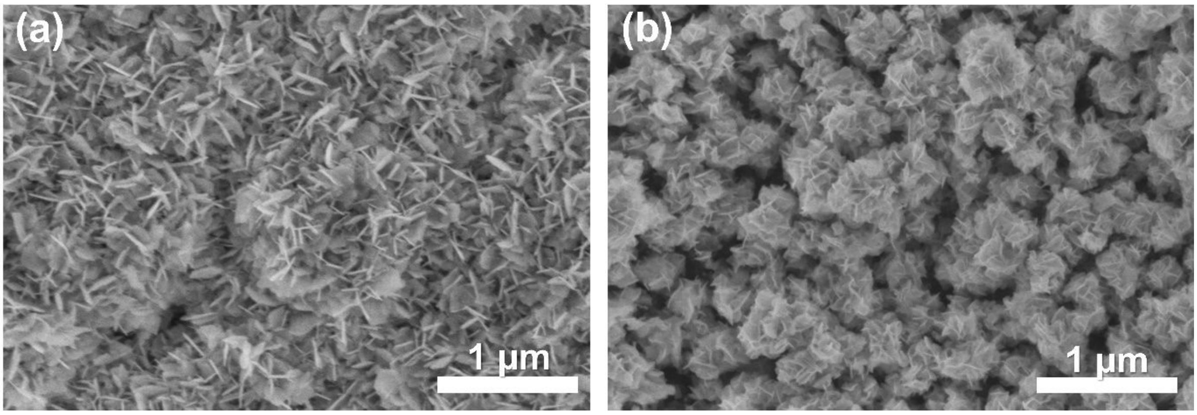

Figure S6. SEM images of BiOI-250 (a) after CV and (b) after reaction at 100 mA cm^{-2}

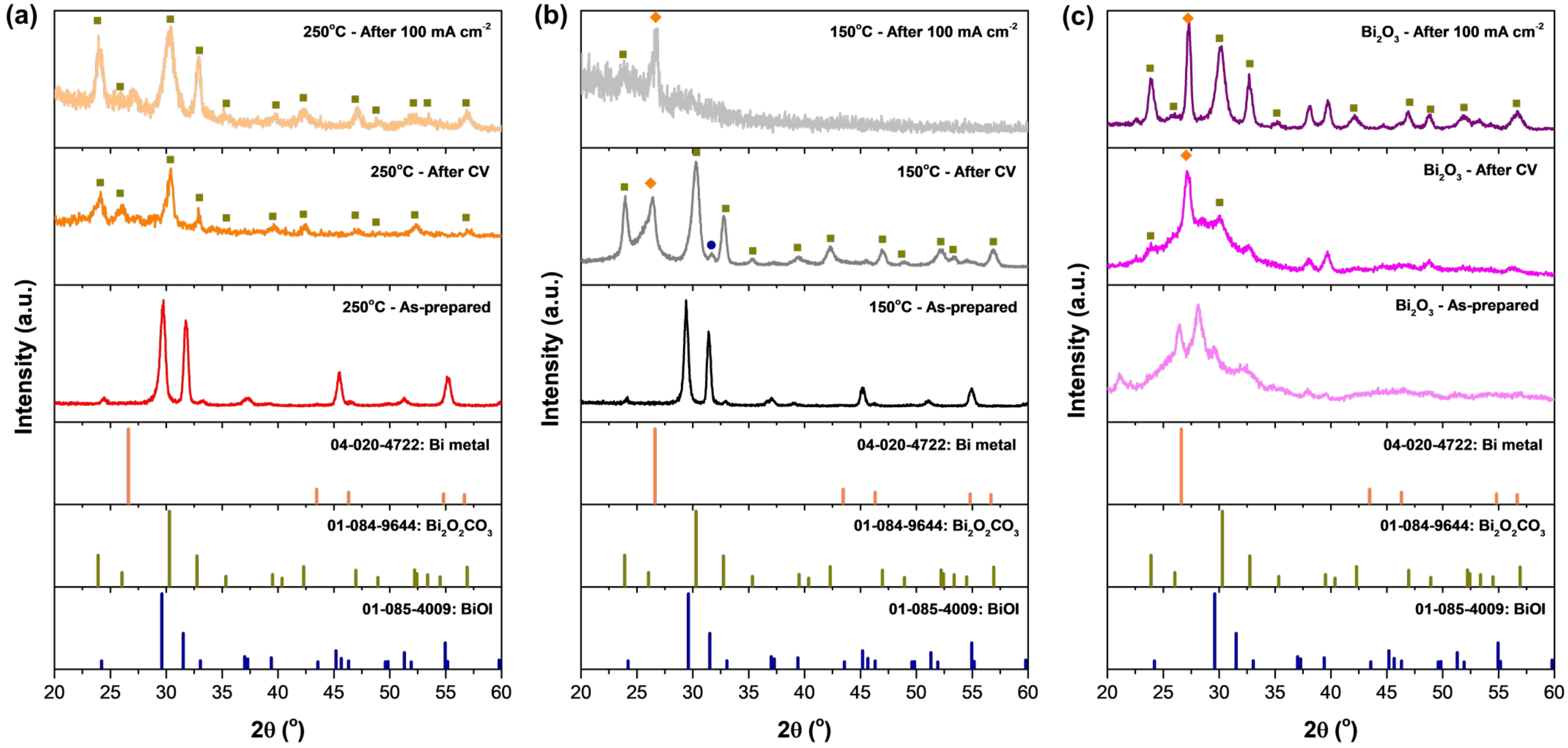

Figure S7. XRD patterns of (a) BiOI-250, (b) BiOI-150, and (b) Bi_2O_3 electrode under varying conditions, including as-prepared, after CV, and after CO_2RR at 100 mA cm^{-2} for 1 hour.

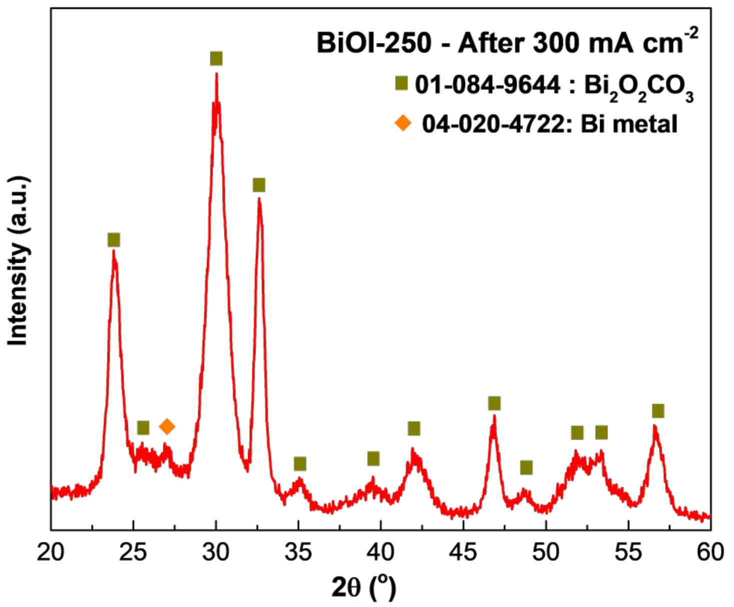

Figure S8. XRD patterns of BiOI-250 after CO_2RR at 300 mA cm^{-2} for 1 hour

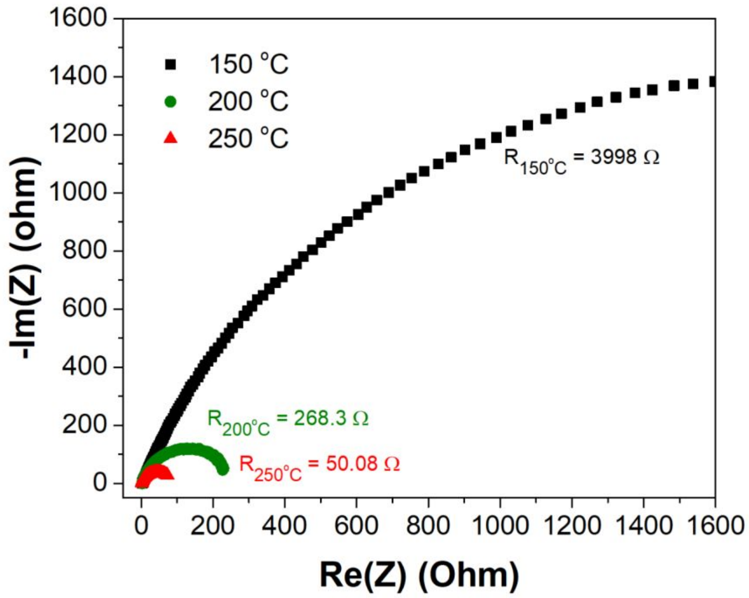

Figure S9. Nyquist plot of fresh BiOI electrodes measured at 0.71 V vs. RHE.

Table S3. XANES and linear combination fit (LCF)

**Sample**	**Composition (%)**			
	**Bi metal**	**Bi_2O_3**	**$Bi_2O_2CO_3$**	**BiOI**
BiOI-150 After CV	11.7	53.3	35.0	
BiOI-150 After reaction	52.7	0.4	46.9	
BiOI-250 After CV	18.8	22.7	32.6	25.9
BiOI-250 After reaction			100.0	

Table S4. EXAFS fitting parameters of BiOI samples

**Sample name**	**Path name**	**CN**	**σ^2**	**R**	**E_0**
BiOI-150 After CV	Bi-O_1	0.95	0.013	1.93	-13.931
	Bi-O_2	2.84	0.007	2.26	
	Bi-Bi	0.95	0.003	3.52	
BiOI-150 After reaction	Bi-O_1	4.08	0.014	2.07	-17.87
	Bi-O_2	8.16	0.011	2.32	
	Bi-Bi	1.02	0.003	3.38	
BiOI-250 After CV	Bi-O_1	1.52	0.006	2.14	-18.13
	Bi-O_2	0.76	0.011	2.56	
	Bi-Bi	0.76	0.008	3.17	
	Bi-I	3.02	0.007	3.42	
BiOI-250 After reaction	Bi-O_1	1.74	0.001	2.23	-8.36
	Bi-O_2	5.21	0.025	2.11	
	Bi-Bi	1.74	0.005	3.53	
